# Maximum penalized likelihood estimation of interspike interval distribution

**DOI:** 10.1186/1471-2202-14-S1-P219

**Published:** 2013-07-08

**Authors:** Ondrej Pokora, Lubomir Kostal

**Affiliations:** 1 Department of Mathematics and Statistics, Faculty of Science, Masaryk University, Brno, Czech Republic; 2 Institute of Physiology, Academy of Sciences of the Czech Republic, Prague, Czech Republic

## 

We address the problem of non-parametric estimation of the recently proposed measures of statistical dispersion of positive continuous random variables. The measures are based on the concepts of differential entropy and Fisher information and describe the "spread" or "variability" of the random variable from a different point of view than the ubiquitously used concept of standard deviation. The maximum penalized likelihood (MPL) estimation [1,2] of the probability density function is applied and a methodology how to estimate the dispersion measures is presented. We illustrate the approach on three standard statistical models describing the neuronal activity.

Frequently, the dispersion of measured data needs to be described. Although standard deviation is used ubiquitously for quantification of variability, such approach has limitations. For example highly variable data might not be random at all if it consists only of "extremely small" and "extremely large" measurements. Although the probability density function, or its estimate provide a complete view, quantitative methods are needed in order to compare different models or experimental results. In a series of recent studies, some alternative measures of dispersion were proposed. However, the estimation of these coefficients from data is more problematic. We present the obtained estimations of the dispersion coefficients based on the MPL method, which extends our previous study [3].
